# Environmental Factors Associated with Larval Habitats of Anopheline Mosquitoes (Diptera: Culicidae) in Metema District, Northwestern Ethiopia

**DOI:** 10.18502/jad.v14i2.3733

**Published:** 2020-06-30

**Authors:** Esayas Aklilu, Mizan Kindu, Araya Gebresilassie, Solomon Yared, Habte Tekie, Meshesha Balkew

**Affiliations:** 1 Department of Biology, Mada Walabu University, Bale-Robe, Ethiopia; 2 Department of Parasitology, School of Medicine, Microbiology and Immunology, MadaWalabu University, Bale-Robe, Ethiopia; 3 Department of Zoological Sciences, Addis Ababa University, Addis Ababa, Ethiopia; 4 Department of Biology, Jigjiga University, Jigjiga, Ethiopia; 5 Aklilu Lemma Institute of Pathobiology, Addis Ababa University, Addis Ababa, Ethiopia

**Keywords:** *Anpheles gambiae* s.l., Larval habitat, Metema, Ethiopia

## Abstract

**Background:**

Malaria is one of the major public health concerns in Ethiopia. Control options available for the management of malaria, include case detection, personal protection and larval source management. Effective control of *Anopheles* larvae largely depends on understanding of the habitats of the vectors. The aims of this study were to identify the breeding habitats of mosquitoes and characterize the larval habitats in Gende Wuha Town in northwestern Ethiopia.

**Methods:**

Different aquatic habitats were sampled and characterized for anopheline larvae from November 2012 to June 2013.

**Results:**

In total, 2784 larvae of *Anopheles* mosquitoes were collected from various breeding habitats. Microscopic identification of the III and IV instars revealed the presence of seven *Anopheles* species. Of the *Anopheles* spp, *Anopheles gambiae* s.l. (80%) was the most predominant species in the study area. Spearman correlation coefficient results also determined that the density of *An. gambiae* s.l. increased significantly with habitat temperature (r= 0.346, p< 0.01). Significantly higher *An. gambiae* s.l. larvae were obtained in non-shaded habitats (z= −3.120, p< 0.05) when compared with shaded habitats.

**Conclusion:**

The current study demonstrated *An. gambiae* s.l., the principal malaria vector in the country, is the predominant species in the larval sampling habitats. It was also noted the importance of edge of stream as larva breeding habitats for this species during the dry season of the year. Therefore, attention should be given for this breeding habitat for control of the vector during dry season.

## Introduction

Malaria is a disease caused by parasitic protozoans of the genus *Plasmodium* and transmitted from person to person by the bites of infected female *Anopheles* mosquito. It is endemic in tropical and subtropical regions and prevalent in more than 91 countries. Nowadays almost half of the world’s population is at risk of the disease, mostly those living in resource limited countries. Despite tremendous efforts have been made to curb malaria, the disease is still a leading cause of morbidity and mortality globally. For instance in 2015, 212 million cases and 429,000 malaria deaths were reported. Ninety percent of all malaria cases and 92 % of deaths occurred in the Sub-Saharan African countries (1, 2).

In Ethiopia malaria is one of major public health concerns. In 2017, 2.6 million cases and 5369 deaths were reported in the country (3). About 60% of the country’s populations live in malarious areas, and 68% of the country’s landmass is favorable for transmission, mainly at altitude below 2000m (4). Transmission of the disease is mainly described by seasonal and predominately unstable. The main transmission follows the major rainy season (June–September) and takes place from September–December while minor transmission happens in April–May immediately after the short rainy season (February–March) (5).

So far, in Ethiopia, 43 species and subspecies of anopheline mosquitoes have been registered (6). Of these, four species of *Anopheles* are linked with malaria transmission, namely, *An. arabiensis*, *An. pharoensis*, *An. funestus* and *An. nili*. The former one is the major vector whereas the rest are secondary vectors (5, 7).

Larvae of *An. arabiensis* breeds in small, temporary, sun-lit water collections created during and after the rains albeit it can also breed in a wide variety of other types of water bodies. Sometimes it also breeds in seasonal breeding habitats that are created by human activity, for instance brick making pits, sand mining pools, human foot and hoof prints of animals, tyre-rut of tractor and other vehicles in agricultural development, drainage and irrigation canals (7, 8).

The larval breeding habitat is important for mosquito population dynamics, due to it is a place where a number of crucial life cycle processes take place such as ovipostion, sub-adults development, and emergence of adult occur (9). Better understanding of larval ecology and delineating of larval habitat attributes in terms of environmental characteristics are indispensible for devising novel malaria vector control strategies (10).

In Ethiopia, a few previous attempts have been undertaken to identify and characterized the breeding habitats of anopheline larva. Teklu et al. (11) pointed out the major vector of malaria prefer to breed in slightly turbid and shallow aquatic habitat in the shoreline of the Koka reservoir, central Ethiopia. In the same general area, Kenea et al. (8) revealed other environmental factors, which determine the relative abundance of *An. arabiensis*. Accordingly, the density of larvae of this species was significantly and inversely associated with aquatic vegetation and water current. More recently, Animut et al. (12) reported that density of larvae of *An. arabiensis* had direct and indirect association with water temperature and depth of larval habitat, respectively in Butajira, south central Ethiopia. However, nothing has been known about species composition of *Anopheles* larvae and their larval breeding habitats in Gende Wuha Town where malaria is one of major public health problems. Hence, the aim of this study was to determine the species composition of *Anopheles* larvae, identify and characterize the larval habitats of anopheline mosquitoes in malaria endemic area of Gende Wuha Town, northwest Ethiopia.

## Materials and Methods

### Study area

The study was conducted in Gende Wuha Town in Metema District, Southwest Gondar. It is located 150km and 860km from Gondar and Addis Ababa, respectively. The Town is located at latitude of 12^o^ 46N and longitude of 36^o^ 24 E and at altitude of 734 meter above sea level (a s l). The rainfall is characterized by a unimodal distribution with annual rainfall that ranges between 500–800mm. The rainy season begins from mid-June and lasts until the end of September. The dry season is much extended and it starts from October and lasts until the end of May. The average day temperature is usually high (25–30 °C) most of the year reaching 40 °C during the months of April–May.

### Larval sampling and processing

Anopheline larvae were sampled fortnightly for eight consecutive months from November 2012 to June 2013. Sampling of anopheline larvae was made from any potential larval breeding habitat that was encountered during the survey. During each survey, a habitat was first visually inspected for the presence of mosquito larvae, and then depending on the size of the breeding habitat ten to twenty samples were taken with a soup ladle (350ml capacity) from each breeding habitat (13). Samplings were al-ways done by the same individual in the morning (9:00–12:00). The *Anopheles* larvae were separated from the culicine larvae based on the position of the larvae to the surface of water. The former larvae were classified as early in-stars (I and II) and late instars (III and IV). The early instars were counted and discarded whereas all the late instars were killed with warm water and preserved in 70% alcohol for species identification based on morphological characters. In the laboratory, each larva was mounted on a glass slide individually in a drop of Hoyer’s medium and covered with a cover slip (14), and species identification was carried out using the key of Gillies and Coetzee (15).

### Characterization of larval habitat

At the same time with larva sampling, different environmental characteristics including turbidity, pH, temperature, speed, distance to the nearest house, length, width, depth, trees nearby to the habitat (shade), presence of aquatic vegetation, presence of algal mat, type of substrate, origin of habitat, habitat permanence of each larva habitat were measured or estimated and recorded. Turbidity was measured by putting water samples in a glass test tube and holding against a white background and was categorized either clean or turbid. Water pH was measured using pH indicator, whereas water temperature was measured using Mercury Thermometer. The speed of aquatic habitat was delineated visually as fast flowing, slow flowing and still (stagnant). Habitat distance to nearest house was estimated visually and classified either greater than or less than 100m. Habitat length, width and depth were measured using measuring tape; shade was recorded as absent or present by observing terrestrial vegetation and/or trees and their branches near to the breeding habitat. Aquatic vegetation was determined visually and classified into four groups (none, emergent, floating debris and emergent and floating debris). The presence or absence of algal mats was visually determined. Type of substrate was categorized as muddy or sandy. Origin of habitat type was classified as either natural or man-made; whereas habitat permanence was estimated visually and classified as permanent, semi-permanent and temporary (10, 16).

### Data analysis

Data analyses were done using SPSS software (version 20 for windows). The association of the larval density of the anopheline mosquitoes with habitats characteristics such as temperature, pH and depth was analyzed using the Spearman correlation coefficient, while association of larval density with some categorical habitat characteristics such as substrate type (stony or muddy), turbidity (low or medium), shade (present or absent) and surface algae (present or absent) were analyzed using the Mann-Whitney U test.

## Results

### Habitat diversity and species composition of *Anopheles* larvae

In the study period, a total of 56 aquatic habitats comprising of edge of stream (n= 33), rain pool (n= 7), puddle (n= 6), spring (n= 5) and streambed (n= 5) were sampled. Fifty-two percent of these habitats were positive for *Anopheles* larvae. A total of 2784 *Anopheles* larvae were collected. Out of these, 64.8% (n= 1804) larvae were early instars (I and II) and the rest (35.2%) were late instars (III and IV). Seven species of *Anopheles* were identified (Table 1). These are *Anopheles gambiae* s.l., *An. pretoriensis*, *An. rufipes*, *An. rhodesiensis*, *An. sergentii macmahoni*, *An. rivulorum* and *An. coustani* group. Of these, *An. gambiae* s.l. is the most preponderate species, which constituted 80% and, followed by *An. pretoriensis* (16.4%). *Anopheles coustani* was the least abundant which comprised 0.1%. Overall, *An. gambiae* s.l. and *An. pretoriensis* jointly accounted for 96.3% of all mosquitoes collected.

**Table 1.** *Anopheles* larvae collected from various breeding habitats in Gende Wuha Town (November 2012–June 2013)

**Habitat**	**No. of habitats**	**Species**							**Total (%)**

		***An. gambiae*** **s.l.**	***An. pretoriensis***	***An. rufipes***	***An.*** ***rhodesiensis***	***An. sergentii macmahoni***	***An. rivulorum***	***An. coustani***	
**Edge of stream**	33	92	74	4	1	4	10	1	186 (18.9)
**Spring**	5	0	60	0	0	0	3	0	63 (6.4)
**Puddle**	6	88	26	0	1	1	0	0	116 (12)
**Stream bed**	5	0	1	10	0	0	1	0	12 (1.2)
**Rain pool**	7	603	0	0	0	0	0	0	603 (61.5)
**Total (%)**	56	783 (80)	161 (16.4)	14(1.4)	2 (0.2)	5 (0.5)	14 (1.4)	1(0.1)	980

### Monthly larval variation

**Fig. 1.**
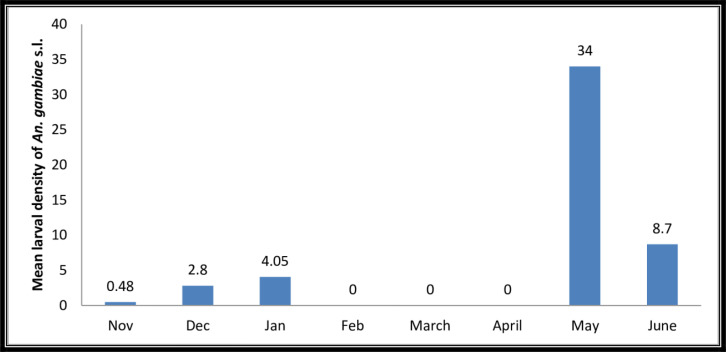
Mean monthly larval density of *An. gambiae* s.l. in the study area (Nov 2012 to Jun 2013)

During the study period, monthly variations in the population density of *An. gambiae* s.l. larval were observed. The highest larval density of *An. gambiae* s.l. was observed in May. From February to April the larval density of *An. gambiae* s.l. was nil as during these time there was no a single breeding habitat of *Anopheles* mosquito (Fig. 1).

### Habitat characteristics associated with larval occurrence

The Spearman correlation coefficient results indicated that the density of *An. gambiae* s.l. had positive and statistically significant with habitat temperature (r= 0.346, p< 0.01), whereas the density of *An. pretoriensis* had negatively associated with habitat temperature notwithstanding the association was very weak and not significant (Table 2). Depth and pH of the habitats had weak positive association with larval density of both species but not significant.

**Table 2.** Anopheline larval density association with different habitat characteristics

**Habitats characteristics**	**Mean±SE**	**Species**			

		***An. gambiae* s.l.**		***An. pretoriensis***	

		**r**	**P-value**	**r**	**P-value**
**Temperature**	25.61±0.7	0.346	0.008^*^	−0.61	0.654
**pH**	6.97±0.1	0.208	0.121	0.168	0 .211
**Depth (cm)**	5.2±0.34	0.058	0.669	0.173	0.055

r-Spearman correlation coefficient,

* Correlation is significant at the 0.01 level

The mean comparison analysis revealed the characteristics of larval habitats and mean densities of anopheline larvae (Table 3). Significantly higher mean densities of *An. gambiae* s.l. larvae were collected from aquatic habitats that had turbid, natural, free of shade and nearby to human dwellings (< 100m). Likewise, significantly higher mean densities of *An. pretoriensis* larvae were obtained from aquatic habitats that had clear, natural, muddy with mats of algae.

**Table 3.** Anopheline larval density association with some of categorical habitat characteristics

**Categorical habitat characteristics**		**Species**					

		***An. gambiae* s.l.**			***An. pretoriensis***		

		**Mean±SE**	**X**^**2**^	**p-value**	**Mean±SE**	**X**^**2**^	**p-value**
**Turbidity**	**Clear**	0.61±0.26	241	0.759	0.36±0.15	181.5	0.049^*^
	**Turbid**	4.55±3.25			0.00±0.00		
**Origin of habitat**	**Natural**	1.62±0.79	173.0	0.23	0.35±0.14	157.5	0.08
	**Man made**	0.08±0.08			0. 0±0.0		
**Presence of algae**	**Present**	1.77±1.28	374.5	0.53	0.56±0.24	265.5	0.002^*^
	**Absent**	1.01±0.57			0.04±0.03		
**Shade**	**Present**	0.00±0.00	207.0	0.02^*^	0.03±0.02	283.0	0.112
	**Absent**	2.00±0.97			0.41±0.17		
**Presence of aquatic vegetation**	**Noon**	1.88±1.1	2.97	0.39	0.41±0.19	1.01	0.798
	**Emergent**	0.98±0.54			0.15±0.09		
	**Emergent and floating**	0.00±00			0.00±0.00		
	**Floating**	0.08±0.06			0.06±0.05		
**Water Current**	**Still**	1.6±0.87	273.0	0.5	0.34±0.15	294.5	0.87
	**Slow flowing**	0.67±0.49			0.15±0.08		
**Type of substrate**	**Muddy**	1.80±1.04	0.71	0.7	0.02 ± 0.02	7.67	0.022^*^
	**Stone**	1.33±1.01			0.48±0.19		
**Habitat permanence**	**Permanent**	0.22±0.08	3.10	0.21	0.34±0.13	4.8	0.08
	**Semi-permanent**	2.29±2.26			0.2±0.12		
	**Temporary**	1.46±0.66			0.32±0.23		
**Distance to the nearest house**	**< 100**	2.11±1.05	317.5	0.2	0.15±0.08	307.5	0.11
	**> 100**	0.11±0.56			0.53±0.29		

Analysis using the Mann Whitney U test indicated that significantly higher *An. gambiae* s.l. larvae density were obtained in non-shaded habitats (z= −3.120, p< 0.05) when compared with shaded habitats. Similar analysis revealed that significantly higher *An. pretoriensis* larvae were collected in habitats that were clear (z= −1.967, p< 0.05), presence of algae (z= −3.034, p< 0.05) and muddy (z= −2.225, p< 0.05) when compared with turbid, absence of algae and stony substrate, respectively.

## Discussion

Of the forty-three known species and subspecies of anopheline mosquitoes in Ethiopia (6), seven of them were found in the present study in larva form. These species were *Anopheles gambiae* s.l., *An. pretoriensis*, *An. rufipes*, *An. rhodesiensis*, *An. sergentii macmahoni*, *An. rivulorum* and *An. coustani* group. The main malaria vector in the country (5, 7) was the most abundant species and followed by *An. pretoriensis*.

Five larval breeding habitat types were identified in the present study area, namely edge of stream, spring, puddle, streambed, and rain pool. Of these habitats, edge of the stream was the most common breeding habitat. During the dry season, this habitat was the main source for anopheline larvae in the area. The same observations were reported in south-central Ethiopia (12) and western Kenya (17). The current study indicated that edge of streams play significant role in maintaining larva of *Anopheles* mosquitoes, especially at the time low or no rainfall occur. As a result, attention should be given for these breeding habitats when larval management strategies are developed to reduce density of anopheline mosquitoes.

In the present study, larval density of *An. gambiae* s.l. increased significantly as with water temperature, as reported previously in central Rift Valley of Ethiopia (11) and south-central Ethiopia (12). These results indicated that water temperature along with other factors might have significant role in larval development and egg hatchability. In contrary to the current observation, Mala et al. (18) reported inverse relationship between the habitat temperature and larval density of *An. gambiae* s.l. in Baringo district of Kenya. Furthermore, Dejenie et al. (19) did not show any association between this environmental parameter and the species in central zone of Tigray. Unlike larvae of *An. gambiae* s.l., larval density of *An. pretoriensis* had negative correlation with water temperature but not statistically significant. The average temperature in water bodies where the *Anopheles* larvae were sampled was 25.61±0.7 °C (ranged from 19 °C to 35 °C). Such temperature is within the range of conducive water temperature in which anopheline larvae survive and develop into adult stage (20).

Mann-Whitney test analysis indicated that the larvae of *An. gamibae* s.l. breed significantly on free of shaded habitats as compared to shaded habitats. Similar result was also reported by Tuno et al. (21) in western Kenya. Contrary to our observation and earlier studies, Mala et al. (18) found that significantly higher larval density of this species in shaded habitats in Baringo district of Kenya.

In the current study, larval density of *An. pretoriensis* was significantly more abundant in the habitats which had algae than without algae. Importantly, previous works showed that the presence of algae is a key factor for the growth of some anopheline species. For instance, Manguin et al. (22) underscored the importance of algae for the presence of *An. pseudopunctipennis* larvae in much of their collection breeding habitats. Likewise, Gimnig et al. (23) demonstrated the strong association between the larval densities of *An. gambiae* s.l and algae in western Kenya, although larvae of *An. gambiae* s.l. were rare or absent in the presence of algae in the present study. Furthermore, Kenea et al. (8) stressed the importance of the algae for the presence of *An. pharoensis* in central Rift valley of Ethiopia.

## Conclusion

In conclusion, our study demonstrated that *An. gambiae* s.l. is the predominant species of *Anopheles* mosquitoes in the larval sampling habitats of Gende Wuha Town. Breeding of this species in the study area is driven predominately by turbid, natural, free of shade and nearby to human dwelling larval breeding sites. It was also noted the importance of edge of stream as larval breeding habitats for *An. gambiae* s.l. during the low precipitation period of the year. The edge of streams may be an important breeding habitat for different *Anopheles* spp and for low transmission of malaria during the dry season. Therefore, control programs designed to contain the transmission of malaria in this particular area through the management of larvae during dry season should take into account the importance of such breeding habitats. However, as the edge streams may not be the only larval breeding habitats in the area, further detailed longitudinal larval surveillance should be carried on in order to make control of the immature stages of *Anopheles* mosquitoes. Moreover, the role of these *Anopheles* species in malaria transmission should be clarified through detailed entomological investigations in the future.
